# Supervised versus autonomous exercise training in breast cancer patients: A multicenter randomized clinical trial

**DOI:** 10.1002/cam4.1851

**Published:** 2018-11-10

**Authors:** Theresa Westphal, Gabriel Rinnerthaler, Simon Peter Gampenrieder, Josef Niebauer, Josef Thaler, Michael Pfob, David Fuchs, Marina Riedmann, Barbara Mayr, Bernhard Reich, Thomas Melchardt, Brigitte Mlineritsch, Lisa Pleyer, Richard Greil

**Affiliations:** ^1^ Department of Internal Medicine III with Hematology, Medical Oncology, Hemostaseology, Infectious Diseases, Rheumatology, Oncologic Center Paracelsus Medical University Salzburg Salzburg Austria; ^2^ Salzburg Cancer Research Institute with Laboratory of Immunological and Molecular Cancer Research and Center for Clinical Cancer and Immunology Trials Salzburg Austria; ^3^ University Institute of Sports Medicine, Prevention and Rehabilitation Paracelsus Medical University Salzburg Salzburg Austria; ^4^ IVth department of Internal Medicine with Hematology and Medical Oncolocy Klinikum Wels‐Grieskirchen, Klinikum Wels‐Grieskirchen Wels Austria; ^5^ mediFIT Wels Medical Fitness and Training Center Wels Austria; ^6^ Department of Internal Medicine 3, Hematology and Oncology Kepler University Hospital Med Campus III Linz Austria; ^7^ Department of Medical Statistics, Informatics and Health Economics Medical University Innsbruck Innsbruck Austria; ^8^ Cancer Cluster Salzburg Salzburg Austria

**Keywords:** breast cancer, early, endocrine therapy, exercise, postmenopausal

## Abstract

**Background:**

There is a well‐known correlation between obesity, sedentary lifestyle, and breast cancer incidence and outcome. The Arbeitsgemeinschaft Medikamentöse Tumortherapie (AGMT) exercise study was a multicenter, randomized clinical trial and assessed the feasibility and efficacy of physical training in 50 breast cancer patients undergoing aromatase inhibitor treatment.

**Methods:**

Postmenopausal, estrogen receptor‐positive breast cancer patients under aromatase inhibitor treatment were randomized 1:1 to counseling and unsupervised training for 48 weeks (unsupervised arm) or counseling and a sequential training (supervised arm) with a supervised phase (24 weeks) followed by unsupervised physical training (further 24 weeks). Primary endpoint was the individual maximum power output on a cycle ergometer after 24 weeks of exercise. A key secondary endpoint was the feasibility of achieving 12 METh/week (metabolic equivalent of task hours per week).

**Results:**

Twenty‐three patients (92%) in the unsupervised arm and 19 patients (76%) in the supervised arm with early‐stage breast cancer completed the study. After 24 weeks, the supervised arm achieved a significantly higher maximum output in watt (mean 132 ±  standard deviation [SD] 34; 95% confidence interval [CI] 117‐147) compared to baseline (107 ± 25; 95%CI 97‐117; *P* = 0.012) with a numerically higher output than the unsupervised arm (week 24 115 ± 25; 95%CI 105‐125; *P* = 0.059). Significantly higher METh/week was reported in the supervised arm compared to the unsupervised arm during the whole study period (week 1‐24 unsupervised: 18.3 (7.6‐58.3); supervised: 28.5 (6.7‐40.1); *P* = 0.043; week 25‐48; *P* = 0.041)).

**Conclusion:**

This trial indicates that patients in an exercise program achieve higher fitness levels during supervised than unsupervised training.

## 1 INTRODUCTION

In 2013, a meta‐analysis of prospective studies including about 63,800 cases suggested that physical activity can significantly reduce the risk of breast cancer depending on the amount of physical activity given by MET (metabolic equivalent task hours per week).^1^ MET has been introduced to facilitate the comparison of different kinds of physical activity. One MET defines a resting metabolic rate given by the amount of oxygen consumed at rest (approximately 3.5 ml O_2_/kg/min = 1.2 kcal/min for a 70 kg person). Two METs require twice the resting metabolic rate, and subsequently, three METs require three times the resting metabolism and so on.^2^

Of note, weight and physical activity do not only play a role in the prevention of breast cancer but influence the course of a current breast cancer disease as well. Overweight or obesity at the time of breast cancer diagnosis seems to be a poor prognostic factor associated with nodal involvement as well as increased disease specific and overall mortality.^3^, ^4^, ^5^ There is an association between changes in body mass index (BMI) after breast cancer diagnosis and breast cancer recurrence as well as waist‐to‐hip ratio and mortality in postmenopausal women with breast cancer.^6^, ^7^

Several cohort studies showed a reduced risk of breast cancer recurrence and new primaries in physical active patients after breast cancer diagnosis.^8^, ^9^, ^10^, ^11^, ^12^, ^13^ A meta‐analysis of epidemiological studies confirmed the positive effect of “adjuvant exercise” with a risk reduction by 21% (HR 0.79; 95%CI 0.63‐0.98, *P* < 0.05),^14^ which is in the range of the risk reduction by adjuvant chemotherapy (rate ratio [RR] 0.79; SE 0.04; χ^2^_1_ = 33.7).^15^

Studies suggest that female breast cancer patients with a moderate physical activity of at least 8.3 METh/week (equivalent to 2‐3 hours of moderate bicycling per week) tend to have a higher quality of life (QoL) and lower incidence of depression and lower all‐cause mortality.^16^, ^17^, ^18^, ^19^, ^20^

Weight gain and reduced physical activity after breast cancer diagnosis is frequent and especially increased under treatment with aromatase inhibitors.^21^ Therefore, guidelines such as the American Society of Clinical Oncology/American Cancer Society recommend nutrition and physical activity counseling for breast cancer survivors and advocate especially strength training for women under adjuvant or hormone therapy.^22^, ^23^ However, adherence to lifestyle behavior recommendation in cancer survivors is low.^24^ Therefore, guided cancer survivor programs are warranted to increase the adherence to physical activity that is of most importance for the individual cancer survivor but also for the healthcare system.

The Arbeitsgemeinschaft Medikamentöse Tumortherapie (AGMT) exercise study aimed to compare the training effect expressed as the individual maximum power output in watt on a cycle ergometer and physical activity calculated as METh/week during unsupervised or supervised exercise training in breast cancer patients during aromatase inhibitor treatment. Additionally, we assessed the compliance of participation in a training program, the QoL, BMI change, and longitudinal change of metabolic markers.

## 2 MATERIALS AND METHODS

### 2.1 Study design and patients

#### 2.1.1 Trial design and inclusion criteria

This exercise study was a multicenter, randomized, parallel group, open‐label clinical trial. Postmenopausal women with hormone receptor‐positive breast cancer irrespective of tumor stage and age with an Eastern Cooperative Oncology Group (ECOG) performance status of ≤2, being able to perform the physical activity program, and without clinical contraindications to exercise were eligible for the study. Patients with uncontrolled heart and lung disease, uncontrolled cardiac arrhythmia, uncontrolled diabetes mellitus or hypertension, uncontrolled pulmonary or cardiac disease, chronic infections or active opportunistic infections, and pathologic ergometer tests were excluded from participation. Patients were required to complete repeated questionnaires on QoL, and lifestyle, including physical activity.

#### 2.1.2 Randomization and overall study design

Patients were stratified according to BMI (≤25 and >25 kg/m^2^) and were randomized 1:1 using permuted blocks. All patients received counseling for nutrition, lifestyle, and physical activity including guidance on endurance training and strengthening exercises. Patients randomized to the unsupervised arm were asked to perform an unsupervised training for 48 weeks. This training included an endurance period of 2.5 hours per week as well as strength training twice a week according to the World Health Organization (WHO) recommendations on physical activity for health.^25^ Patients randomized to the supervised arm underwent a supervised training program of physical activity for 24 weeks followed by unsupervised exercise training for further 24 weeks. The supervised training comprised of 45 minutes of stationary cycling and 30 minutes of controlled resistance training twice a week. All interventions were provided free of charge. Patients enrolled in Salzburg (n = 39) trained at the University Institute of Sports Medicine, Prevention and Rehabilitation (Salzburg, Austria), whereas patients from Wels (n = 9) or Linz (n = 2) were supervised at the hospital medical fitness center. In addition, patients were asked to perform 30 minutes of walking or cycling at home twice per week at their individually recommended training heart rate. In detail, endurance exercise training was performed as high‐intensity interval training on cycle ergometers (Ergoline®, Bitz, Germany) under ECG control. Training was performed at 70% of the maximal heart rate, and all training sessions were electronically recorded. Supervised resistance training comprised of ten muscle endurance strength training exercises, which were performed on weight‐lifting machines: Latissimus pull down, back extension, chest press, leg press, leg extension, leg flexion, dips, rowing, abdominal crunches, cable pull. During the first strength training session, the ten‐repetition maximum (10‐RM) was assessed for each exercise. Muscle endurance training was carried out at intensities calculated to initially permit not more than 30 repetitions to failure. Whenever more than 30 repetitions could be performed, weight was increased. One set of each exercise was carried out per training session.

#### 2.1.3 Informed consent and independent ethics committee

Approvals from independent Ethics committees in Salzburg and Upper Austria (415‐E/1290 and C‐39‐12) were obtained before the beginning of the study. Written informed consent was obtained from all participants.

### 2.2 Objectives

The primary endpoint of the study was the individual maximum power output in watt (P_max_) on a cycle ergometer after 24 weeks of supervised or unsupervised training. Secondary objectives were the determination of the feasibility of achieving 12 METh/week on an outpatient basis. The threshold of 12 METh/week was selected based on the findings from the women’s health initiative study, where women with a physical activity of 9 or more METh/week after breast cancer diagnosis had a lower all‐cause mortality (HR = 0.67; 95% CI 0.46‐0.96).^20^ Twelve METh/week are equal to three hours of moderate bicycling (10 mph = 16.1 km/h) or 1.5 hours of running (5 mph = 8km/h).^18^ Furthermore, the determination of adherence and compliance of patients to nutritional, lifestyle, and physical activity counseling and to supervised physical exercise, respectively, was assessed.

Further secondary objectives were the evaluation P_max_ on a cycle ergometer after 48 weeks. Quality of life was assessed with the EORTC QLQ‐C30 questionnaire version 3, developed to assess the QoL of cancer patients, and the EORTC QLQ‐BR23 module, a breast cancer‐specific questionnaire.^26^, ^27^ Overall, QoL status was assessed by the “Global health status score,” based on patient’s answers to questions 29 and 30 of the EORTC QLQ‐C30 questionnaire. Furthermore, lifestyle, sport habits, BMI, body fat analysis by caliper measurement,^28^ and waist‐to‐hip ratio were recorded.

At baseline and weeks 12, 24, 36, and 48, blood pressure was measured and blood was obtained to assess complete blood count, metabolic parameters (glucose, cholesterol, triglyceride, HbA1c, leptin, insulin), immune globuline levels (IgG, IgA, IgM), tumor markers (CEA, CA15.3), homocystein, TNF‐alpha, c‐peptide, IGF‐1, IGF‐1BP, sex hormone‐binding globulin, and hormone levels (FSH, LH, progesterone, testosterone, E2). Additionally, electrolytes, liver function parameters, thyroid hormones, iron levels, and cardiac enzymes were taken at baseline (see Table 1).

**Table 1:** Study assessment

Parameter	Baseline	Week 12	Week 24	Week 36	Week 48
Medical history	x				
QoL questionnaire	x	x	x	x	x
Co‐medications	x	x	x	x	x
Diary	x	x	x	x	x
EKG	x	x	x	x	x
Echocardiography	x				
Ergometry with lactate measurement	x		x		x
Blood pressure	x	x	x	x	x
BMI	x	x	x	x	x
Body fat analysis (caliper)	x	x	x	x	x
Weight	x	x	x	x	x
Blood count, metabolic parameters (glucose, cholesterol, triglyceride, HbA1c, leptin, insulin), immune globuline levels (IgG, IgA, IgM), tumor markers (CEA, CA15.3), homocystein, TNF‐alpha, c‐peptide, IGF‐1, IGF‐1BP, sex hormone‐binding globulin, and hormone levels (FSH, LH, progesterone, testosterone, E2).	x	x	x	x	x
Electrolytes, liver function parameters, thyroid hormones, iron levels, and cardiac enzymes	x				

### 2.3 Patient monitoring and safety

At baseline, the eligibility for exercise training was assessed by echocardiography, ECG, an ergometry with lactate measurement and blood pressure measurement. Participants in the supervised arm were continuously monitored during the supervised endurance exercise program *via* ECG. Furthermore, a qualified sport scientist was present at all training times. Facilities for resuscitation and first aid were provided by the hospital in which the exercise program was performed. MET values were self‐reported using patient diaries and were analyzed as the average METh/week during the supervised (weeks 1‐24) and unsupervised study phase (weeks 25‐48).

### 2.4 Statistical analysis

Regarding our primary endpoint (P_max_ at week 24), we postulated an increase by supervised or unsupervised training of at least 20% compared to baseline, according to Courneya et al.^29^ Courneya et al included anemic solid tumor patients under darbepoetin in a randomized interventional exercise study and observed a postinterventional standard deviation of 30.45 watts. Since our study population was predicted to be a more homogenous group of female breast cancer patients, we assumed our standard deviation to be lower. We thus expected a mean P_max_ of 89 watts, as observed postinterventional by Courneya et al, but with a lower standard deviation by 20% yielding a value of 24.36 watts. Based on these assumptions, a sample size of 35 in each group was calculated to have 80% power to detect an absolute difference of 16.56 watts in P_max_ (according to a standard deviation of 24.36) with a 0.05 two‐sided significance level using a two‐group *t* test. To account for dropouts and for the likely necessity to use a nonparametric statistical test, we assumed that 40 patients per group would have to be randomized. With the actual sample size of 42 patients, there was 80% statistical power to detect a difference of 22 watts.

All variables were presented using descriptive summary statistics (continuous data: mean, standard deviation, median, range, 95% confidence interval; categorical data: sample size, absolute and relative frequency). Based on the distribution of the analyzed continuous variables, either a *t* test for independent samples or a Mann‐Whitney U test was applied to compare variables between study arms at the different timepoints. Categorical variables were compared with chi‐square or Fisher’s exact test, respectively. Statistical tests were generally two‐sided with a significance level of 5%. Statistical analyses were performed using IBM® SPSS® statistics software, version 21.

The primary endpoint (P_max_) was compared using three different tests, that is, (a) ANCOVA for repeated measurements for the intention‐to‐treat population with imputation of missing values according to the last observation carried forward (LOCF) principle, (b) a mixed effects model without imputation (per‐protocol analysis), and (c) *t* test at single timepoints. However, except two patients (withdrew 6 months before the exercise evaluation), all patients dropped out after baseline measurement. Because no exercise training data are available from these patients, they were not included into final analysis of the primary endpoint.

Spearman’s correlation coefficients (r_s_) were computed to explore the associations between the P_max_, METh/week, BMI, and global health score.

## 3 RESULTS

### 3.1 Patient recruitment

**Figure 1:**
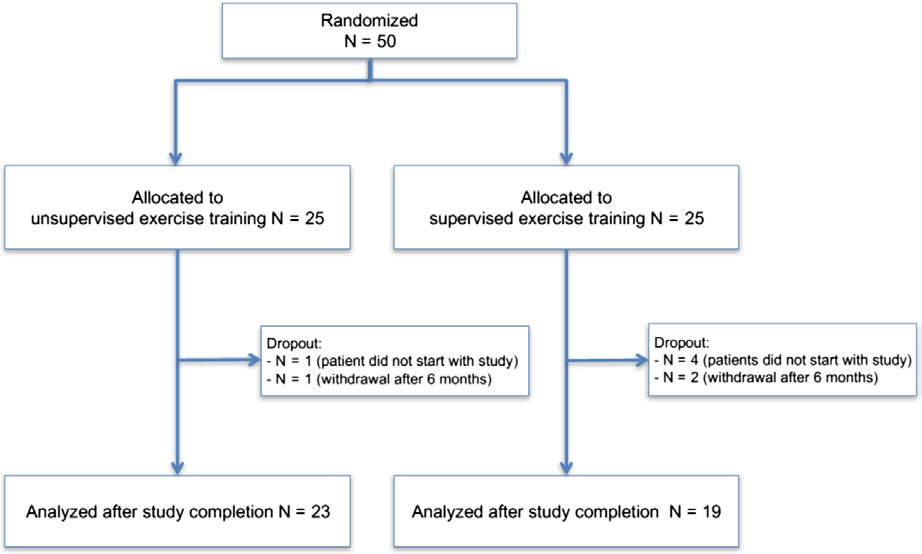
CONSORT diagram

Patients were recruited in three Austrian centers. Due to slow recruitment, the trial was closed prematurely after recruiting 50 of 80 planned patients (unsupervised: 25, supervised: 25). Overall, 23 patients in unsupervised and 19 patients in supervised completed the study (see Figure 1).

### 3.2 Patient characteristics

The clinical and baseline characteristics were well balanced (Table 2). There were no major differences regarding age, weight, and BMI (stratified) between the unsupervised and supervised arm. All patients in this study had estrogen receptor (ER)‐positive breast cancer. Most patients had Her2/neu‐negative status (unsupervised 76%, supervised 84%).

**Table 2:** Baseline characteristics

		Unsupervised N (%) [range]	Supervised N (%) [range]	*P* (*t* test)
Age (years)	Mean Range	60.76 [49‐74]	61.0 [48‐81]	0.846
Weight (kg)	Mean Range	74.6 [47.0‐111.0]	73.712.3 [55.0‐108.0]	0.818
BMI	Mean Range	27.81 [18.83‐45.61]	27.23 [19.96‐37.81]	0.707
Body fat (%)	Mean Range	36.4 [20.3‐48.2]	38.7 [29.7‐46.7]	0.239
Waist‐to‐hip ratio	Mean Range	0.87 [0.73‐0.98]	0.91 [0.76‐1.15]	0.134
Her2/neu Status	Positive	5 (20)	4 (16)	0.725 (Fisher’s exact test)
	Negative	19 (76)	21 (84)	
	Missing	1 (4)	0 (0)	
Estrogen receptor	Positive	25 (100)	25 (100)	1.0
	Negative	0 (0)	0 (0)	
Progesterone receptor	Positive	24 (96.0)	21 (84.0)	0.349 (Fisher’s exact test
	Negative	1 (4)	3 (12)	
	Missing	0 (0)	1 (4)	
Neoadjuvant chemotherapy(+ trastuzumab in Her2neu‐positive patients)	No	21 (84)	24 (96)	0.349 (Fisher’s exact test)
	Yes	3 (12)	1 (4)	
Adjuvant chemotherapy (+ trastuzumab in Her2neu‐positive patients)	No	14 (56)	17 (68)	0.561 (Fisher’s exact test)
	Yes	11 (44)	8 (32)	
Aromatase therapy (drugs)	Anastrozole	20 (80)	22 (88)	0.702 (chi‐square test)
	Letrozole	5 (20)	3 (12)	

All patients had early‐stage breast cancer, except one patient in the unsupervised arm who had hepatic metastatic disease at study entry. The average time between cancer diagnosis and study inclusion was 29.1 (1‐171) months in the unsupervised arm and 22.3 (1‐133) months in the supervised arm with no significant differences between the two arms. Also, there were no significant differences between times from surgery to study inclusion (unsupervised: median 12 months, range 0‐171 months; supervised: median 8 months, range 0‐86 months; *P* = 0.322).

The study was not powered to compare survival data between the arms. During the study, two patients in unsupervised and one patient in supervised had a progression of the disease. There were no deaths recorded during study phase.

### 3.3 Maximum power output

**Figure 2:**
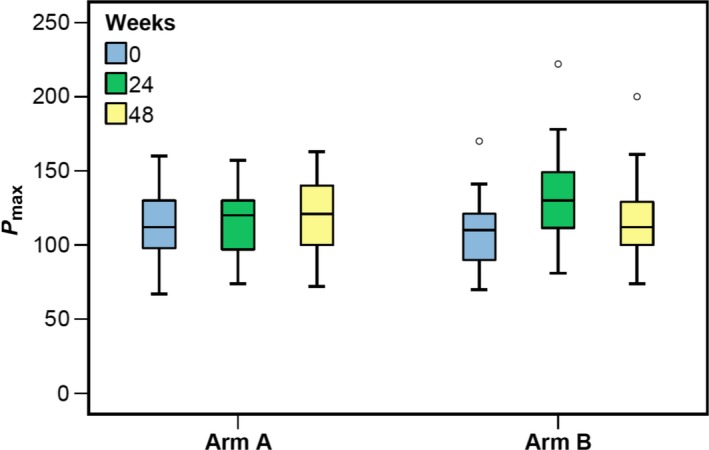
Maximum power output in watt boxplot. Boxplot of maximum power output (P_max_) in watt by study arm (unsupervised exercise training and supervised exercise training) and time: gray color: week 0, green color: week 24; yellow color: week 48

**Figure 3:**
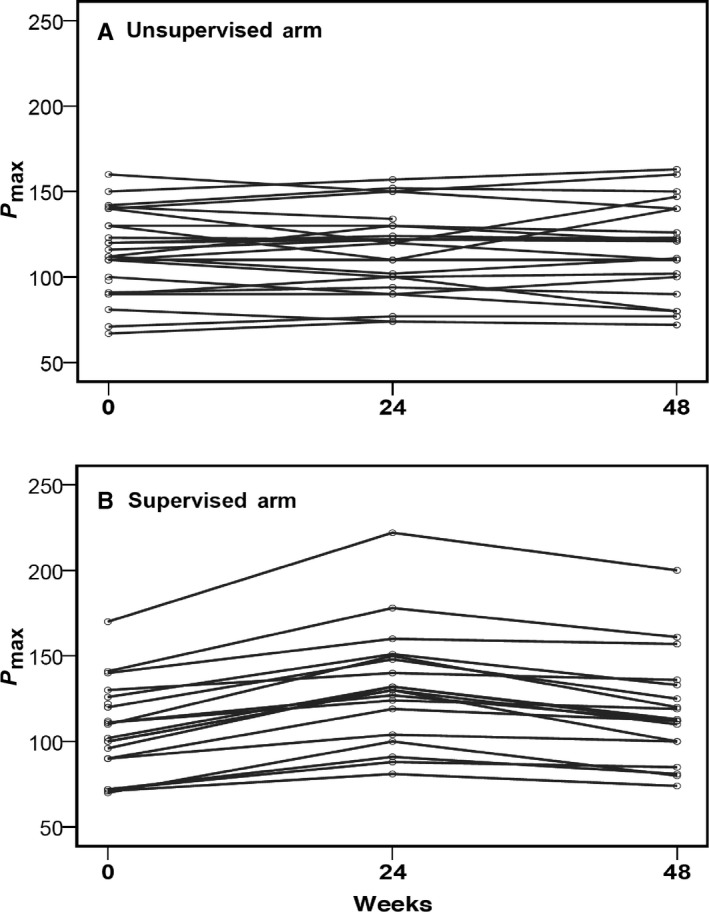
Maximum power output (watt) single patients (unsupervised and supervised) Maximum power output P_max_ (watt) single patients (unsupervised and supervised)

Between baseline (week 0) and week 24, power output (P_max_) improved significantly in the supervised arm by 23% (mean P_max_ week 0: 107 ± 25 W, 95%CI 97‐117; week 24: 132 ± 34 W, 95%CI 117‐147; *P* = 0.012), whereas no difference was found in the unsupervised arm (mean P_max_ week 0: 114 ± 24 W, 95%CI 104.6‐132.4; week 24: 115 ± 25 W, 95%CI 105‐125; *P* = 0.933) (see Table 3 and Figure 2, 3). Comparing both arms at week 24, we observed higher watts in the supervised arm (corresponding to a difference of 17 W or 14.8%) than in the unsupervised arm that failed to reach significance (*P* = 0.059).

**Table 3:** Maximum power output in watt

		Unsupervised training				Supervised exercise training						*P* (*t* test)^*^
		Mean	SD	Range	95% CI	N	Mean	SD	Range	95% CI	N	
P_max_ in watt	Baseline	114.2	24	67‐180	104.1‐124.2	25	107.2	25	70‐170	96.3‐118.1	23	0.337
	Week 24	114.8	25	74‐157	104.4‐125.1	24	132.0	34	81‐222	115.8‐148.1	19	0.059
	Week 48	116.7	27	72‐163	104.9‐128.5	22	117.4	31	74‐200	1102.4‐132.3	19	0.940

P_max_ in watt	Unsupervised training		Supervised training	
	∆ Watt	*P*	∆ Watt	*P* **
P_max_ in watt Week 1: week 24	0.6	0.933	24.7	0.012
P_max_ in watt Week 1: week 48	2.5	0.731	10.2	0.252

SD, standard deviation; P_max_, maximum power output in watt; 95% CI, 95% confidence interval; ∆ Watt, difference in watt between given timepoints

*P* values represent differences between study arms at the same timepoints.

a *P* values represent differences between different timepoints within the same study arms

Analyzing the changes in the power‐to‐weight ratio (∆W/kg), patients in the supervised arm (mean week 0‐week 24: 0.36, SD ± 0.19) showed a significantly greater increase than those in the unsupervised arm (mean week 0‐week 24: −0.05, SD ± 0.19) (*P* < 0.001).

At the end of the study, that is, after discontinuation of exercise training for 24 weeks, we still observed higher P_max_ values compared to baseline in both arms (unsupervised: mean week 48: 117 ± 27 W, 95%CI 106‐128; *P* = 0.731; supervised: mean week 48: 117 ± 31 W; 95%CI 103‐131; *P* = 0.252), with no significant differences between arms (*P* = 0.940). Over the course of the study, however, the gain in maximal power per kg body weight (∆W/kg) was significantly greater in supervised patients (mean week 0‐week 48: 0.18, SD 0.15) than in unsupervised patients (mean week 0‐week 24 unsupervised: −0.01, SD 0.18) (*P* = 0.001).

### 3.4 Physical activity

According to patients diaries, the majority of patients in both arms reported 12 METh/week (72.7% unsupervised, 89.5% supervised, *P* = 0.177; see Table 4) during the whole study period. Nearly all patients reported 12 METh/week during at least one evaluation period (ie, week 1 to 24 weeks; 24 to 48 weeks; unsupervised 72.7%, supervised 94.7%; *P* = 0.062). There was a significantly higher number of patients reporting 12 METh/week during the supervised training phase (supervised: 94.7% vs unsupervised: 68.2%; *P* = 0.032). Also, a trend was seen toward more supervised than unsupervised patients reporting 12 METh/week during the unsupervised training phase (89.5% vs 63.6%; *P* = 0.055). When analyzing the reported METh/week, supervised patients documented significant higher values during both study periods (week 1 to week 24: unsupervised, 18.3 (7.6‐58.3); supervised: 28.5 (6.7‐40.1), *P* = 0.043; week 25 to week 48 unsupervised: 20.8 (9.2‐49.4); supervised: 21.6 (1.4‐44.8), *P* = 0.041). During supervised training, a significant correlation between METh/week and P_max_ was observed (week 1 to week 24: *r*_s_ = 0.585, *P* = 0.000058). Also, a moderate correlation was observed between METh/week and P_max_ for the unsupervised period (week 25 to week 48: *r*_s_ = 0.332, *P* = 0.037) as well as for the whole study period (*r*_s_ = 0.431, *P* = 0.006).

**Table 4:** Physical activity (METh/week)

Outcome parameters (patients achieving 12 METh/week)			Unsupervised training		Supervised training			*P* (Fisher’s exact test)
			N	%	N	%		
METh/week, 1‐24 weeks	Patients achieving 12 METh/week	15 (22)		68.2%	18 (19)	94.7%	0.032	
METh/week, 25‐48 weeks	Patients achieving 12 METh/week	14 (22)		63.6%	17 (19)	89.5%	0.055	
METh/week, 1‐48 weeks	Patients achieving 12 METh/week	16 (22)		72.7%	17 (19)	89.5%	0.177	

METh/week, metabolic equivalent of task per week; N, number of patients

### 3.5 Body mass index and weight

There was no significant difference regarding change in weight, BMI, and body fat over time between both arms (BMI: unsupervised, week 0: 27.8 kg/m^2^ [95%CI: 25.3‐30.2]; week 48: 27.2 kg/m^2^ [95%CI: 24.5‐29.8]; *P* = 0.739; supervised: week 0: 27.2 kg/m^2^ [95%CI: 25.5‐28.9]; week 48: 27.7^ ^kg/m^2^ [95%CI: 25.5‐29.9]; *P* = 0.738). There were no significant changes of the waist‐to‐hip ratio within arms (unsupervised week 24 vs 0: *P* = 0.89, week 48 vs 0: *P* = 0.52; supervised week 24 vs 0: *P* = 0.9, week 48 vs 0: *P* = 0.48) and no significant differences between arms at all three points in time (week 1: *P* = 0.13, week 24: *P* = 0.807, week 48: *P* = 0.63).

### 3.6 Blood pressure and laboratory parameters

There were no significant changes between arms regarding the tested laboratory parameters and blood pressures over time (see Supporting Information Table S1). Using mixed effects modeling also, no significant differences were observed over time within arms with regard to systolic and diastolic blood pressure levels, as well as plasma levels of cholesterol, high‐density lipoprotein (HDL), sex hormone‐binding globulin (SHBG), insulin, and insulin‐like growth factor 1 (IGF‐1). Mean and median levels of all investigated laboratory parameters were within the normal range. There were no significant differences regarding FSH, LH, progesterone, and testosterone between arms. Unfortunately, E2 values were not analyzable due to changes in laboratory equipment.

### 3.7 Quality of life

There were no significant differences between both study arms regarding QoL functional and symptom scales of the QLQ‐C30 questionnaire. However, there were significant improvements between baseline (week 0) and week 48 for upper extremity symptoms (*P* = 0.006) and breast symptoms (*P* = 0.015) according to the QLQ‐BR23 module. There were no significant changes in the Global Health Status, neither between weeks 0 and 24 nor between weeks 0 and 48. This was true in both study arms (unsupervised week 24 vs 0: *P* = 0.89, week 48 vs 0; *P* = 0.52; supervised week 24 vs 0: *P* = 0.90, week 48 vs 0; *P* = 0.48). Patients who achieved 12 METh/week had a slightly higher Global Health Status score, but the difference was not statistically significant (*P* = 0.248).

## 4 DISCUSSION

To our knowledge, the AGMT exercise trial is the first randomized study assessing the feasibility of supervised and unsupervised training in breast cancer patients on endocrine therapy. After 24 weeks of supervised training, maximum power output was significantly increased in the supervised arm by 25 watts (23%), which is above training effects reported by comparable studies.^29^ In the unsupervised arm, maximum power output remained essentially unchanged (0%). Comparison between both arms revealed a trend toward greater maximum power output in the supervised arm as compared to the unsupervised arm. Furthermore, there was a significant difference between the changes in the watt‐per‐weight ratio at week 24 in the supervised compared to the unsupervised group (0.36 ∆W/kg vs −0.05 ∆W/kg; *P* < 0.001). This parameter is more sensitive than power output alone since weight acts as a co‐founder in training that is eliminated by comparing watt/kg.

At week 48 (24 weeks after discontinuation of exercise training), there was a numerically higher output in both arms compared to baseline (unsupervised 117 watts, *P* = 0.731; supervised 117 watts *P* = 0.252) without a significant difference between both arms (*P* = 0.940), but still a significant difference in the ∆W/kg (unsupervised: 0.18 vs supervised: 0.01; *P* = 0.001). Even though the supervised training group did not further improve their exercise capacity during the unsupervised training period, they still reported significantly higher activity levels.

Our results are in line with those published by Courneya et al who observed a postinterventional increase of 14 watts (17%) maximum power output compared to baseline and a significant difference of 12 watts (15%) compared to the nonexercise group after 12 weeks of training.^29^ In contrast to our study, this trial enrolled 26 patients with different solid tumors (including 15 breast cancer patients) who were treated with darbepoetin alpha because of anemia. In our study, patients achieved a higher maximum power output at baseline compared to the study by Courneya et al. Also, the longitudinal increase of maximum power output during study intervention was higher in the AGMT exercise study.

Various cohort studies with breast cancer patients in an adjuvant setting described a continuous inverse correlation between increasing patient activity and decreasing mortality from breast cancer.^8^, ^13^, ^30^ An activity of more than 7.5 METh/week seems to be required in order to reduce breast cancer‐specific mortality, whereas physical activity >21 METh/week showed no additional effect.^9^, ^11^, ^12^ Prospective randomized trials, however, are still missing. Since the AGMT exercise study focused on the feasibility of supervised vs unsupervised training, our trial was not powered to compare survival outcomes. However, since most of the patients in both arms reported having achieved 12 METh/week during the study period (72.7% and 89.5%), no differences in risk reduction by the two interventions on recurrence rate and mortality are expected.

A study of healthy never‐smokers found that the risk of cancer death is significantly increased (hazard ratio [HR] 1.12) with a body mass index (BMI) between 27.5 and 29.9 rising steadily to a HR of 1.7 for those with a BMI of 40 to 49.9.^31^ Premenopausal patients gaining more than 6 kg within 60 weeks after breast cancer diagnosis are 50% more likely to relapse and 60% more likely to die from cancer than patients who do not gain any or even lose weight.^32^ Regarding BMI, there were no significant changes observed in both study arms. In contrast, supervised exercise training in cancer patients led to body fat reduction in an intervention trial published by Grabenbauer et al.^33^ However, in this study, no patients with an already regular physical exercise training were included and the change in BMI was driven by patient <50 years old (average age 49 years compared to about 60 years in our study). Exercise, food intake, and weight loss are closely related and influence each other.^34^, ^35^ We did not assess food habits in this study. However, the primary endpoint “maximum power output on a bicycle” should be independent from food habits and might help to discriminate between food and exercise influence in further studies.

In our study, there were no significant differences between the study arms regarding blood pressure levels and the tested laboratory parameters, respectively. In addition, no significant changes over time in systolic and diastolic blood pressure levels, cholesterol, high‐density lipoprotein, sex hormone‐binding globulin, insulin, and IGF‐1 were observed. Despite the fact that the mean BMI at baseline was in the range of overweight (unsupervised: 27.8, supervised: 27.2), average blood levels of insulin, glucose, inflammatory markers, and free fatty acids were within the normal range. These factors, cytokines, free fatty acids, leptin, higher blood levels of insulin, and glucose are associated with cancer development due to direct effects on oncogenic signaling pathways and indirect effects on tumor microenvironment by inflammatory cells.^36^ Furthermore, it is known that a combination of dietary weight loss and exercise training induces a favorable development of these parameters in breast cancer patients.^37^, ^38^, ^39^ Whether a change of these markers after breast cancer diagnosis effects outcome has not been shown yet.

In our study, there were no significant differences in the quality of life between both arms. However, overall, patients reported less arm symptoms (*P* = 0.006) and breast symptoms (0.015). This is in keeping with other reports that describe poorer quality of life after cancer treatment and that arm symptoms or impaired general functioning can persist.^40^ However, in this study, the power to detect significant changes in quality of life was low, so the results should be interpreted with caution.

Besides the training effect itself, a key element is how to motivate patients to participate in studies and adhere to exercise training recommendations. Without supervised aspects, only a minority of breast cancer survivors (37% in breast cancer) adhere to physical activity recommendations after diagnosis.^24^ Exercise programs can increase adherence rates to 46% (resistance training) and even up to 90% beyond six months in aerobic exercise training.^33^, ^41^, ^42^ Web‐based programs might offer an alternative to supervised center‐based training.^43^ The difficulties with recruitment and high dropout rate in the beginning of the supervised phase can be attributed to long access ways to the training center and motivation aspects. Center‐based exercise programs might increase participation rates, but are more cost‐intensive, whereas home‐based programs are more comfortable to access but might lower adherence rates.

In conclusion, our findings indicate that patients achieve higher fitness levels during supervised training compared to unsupervised training. Patients with supervised training reported significantly higher METh/week not only during the supervised but also in the following period, where both arms only received exercise counseling. In addition, our study showed that almost all mobile and motivated early breast cancer patients can at least temporarily achieve the goal of 12 METh/week irrespective of the kind of training instruction. Nonetheless, meaningful adherence to exercise prescription and an improvement of exercise capacity in this cohort seem only possible with supervised training. For some patients, decentralized training possibilities might be helpful to access training programs but a key question is how to make adhere to them and how pay for them.

## CONFLICT OF INTEREST

The authors declare no conflict of interest.

## AUTHOR CONTRIBUTIONS

R.G. and L.P. planned, coordinated, and conducted the trial. T.W., G.R., S.G., and R.G. drafted and prepared the manuscript. M.P. is the trial statistician. J.N., T.M., B.ML., D.F., B.MA, B.R., J.T., and M.P. participated in designing and conducting the trial. All authors read and approved the final manuscript.
