# Supplementary material for: Supervised versus autonomous exercise training in breast cancer patients: A multicenter randomized clinical trial
Source: Cancer Med. 2018 Nov 10;7(12):5962–72. doi: 10.1002/cam4.1851 (PMC6308077; doi:10.1002/cam4.1851)
Supplement: Supplementary file 1 [file CAM4-7-5962-s001.docx]

**Supplementary information for**

**Supervised versus autonomous exercise training in breast cancer patients:** **a multicenter randomized clinical trial**

*Theresa Westphal, Gabriel Rinnerthaler et al.*

# Endurance training

In detail, endurance exercise training was performed as high intensity interval training on cycle ergometers (Ergoline®, Bitz, Germany) under ECG control. Training was performed at 70% of the maximal heart rate, and all training sessions were electronically recorded. Supervised resistance training comprised of ten muscle endurance strength training exercises, which were performed on weight lifting machines: Latissimus pull down, back extension, chest press, leg press, leg extension, leg flexion, dips, rowing, abdominal crunches, cable pull. During the first strength training session, the ten-repetition maximum (10-RM) was assessed for each exercise. Muscle endurance training was carried out at intensities calculated to initially permit not more than 30 repetitions to failure. Whenever more than 30 repetitions could be performed, weight was increased. One set of each exercise was carried out per training session.

# Study protocol

All patients were screened for their physical ability to complete the interventional exercise program. Only patients capable and willing to take part in this study and meeting all inclusion and exclusion criteria (listed below) were randomized. Randomization was stratified according to BMI. All patients received identical counselling for ideal nutritional / life-style / physical activity.

Patients randomized to Arm 2 will additionally underwent a controlled and observed program of physical activity for a period of 6 months. Thereafter the patients were expected to adhere to a comparable, unobserved exercise program at home.

## Selection of Study Population

### Inclusion Criteria

Patients had to fulfill all criteria listed below prior to enrolment in the study:

- Signed informed consent
- Postmenopausal women with hormone receptor positive breast cancer (as defined by HR >= ++ and/or PR >= ++) who are treated with aromatase inhibitors
- Patients must be able and willing to fill out repeated questionnaires on QOL, lifestyle and sports habits, as well as to adhere to the physical activity program
- Patients must fulfill all screening criteria and be deemed fit, well and able to perform the physical activity program
- ECOG performance status <= 2
- all age groups

### Exclusion Criteria

Patients meeting one or more of the exclusion criteria listed below were not eligible for study participation and weren´t enrolled:

- ECOG >= III
- Pathologic ergometry
- Handicapped patients who are unable to perform ergometry
- Uncontrolled diabetes mellitus
- Uncontrolled hypertension
- COPD > II
- Active opportunistic infection
- NYHA heart failure III or IV
- Relevant, untreated coronary heart disease
- Uncontrolled cardiac arrhythmia
- Active HIV infection
- Active hepatitis B infection uncontrolled by virostatics
- Active hepatitis C infection that requires interferon treatment
- Other diseases with contraindications for physical activity
- Psychiatric or organ dysfunction which would compromise the patient’s safety in the opinion of the investigator, or which could interfere with data interpretation
- Laboratory abnormalities:
  - ALT or AST > 4 times upper normal level
  - Creatinine > 3.5 mg/dl

### Patient safety

Participants in Arm 2 were continuously monitored during the observed/controlled exercise program via heart rate monitoring. Furthermore, a qualified personal trainer was present at all times. Facilities for resuscitation and first aid were provided by the university hospital in which the exercise program was performed.

### Randomization

Randomization is planned on a 1:1 basis for 80 patients in each of the two groups:

- Arm 1: Control Arm
- Arm 2: Intervention Arm

Patients were randomized using permuted blocks and stratified for patient`s body-mass index. For stratification two groups of patients were formed:

- Strata 1: Patients with a body-mass index smaller or equal than 25kg/m²
- Strata 2: Patients with a body-mass index greater than 25kg/m²

Inclusion and exclusion criteria were checked before assignment of the next available randomization number. The identification code list identifying the patient's name, patient number and randomization number were kept by the investigator at the study site.

Figure 1S: Study design

## Flow chart of assessments and procedures

Efficacy and safety variables as well as laboratory tests are described in Tables 1S and 2S.

| Parameter | Screening | 3 months | 6 months | 9 months | 1 year |
| --- | --- | --- | --- | --- | --- |
| Screening | | | | | |
| Medical history | x |  |  |  |  |
| QOL questionnaire | x | x | x | x | x |
| Life style profile questionnaire | x | x | x | x | x |
| Sports profile questionnaire | x | x | x | x | x |
| Depression questionnaire CES-D 20 | x | x | x | x | x |
| Co-medications | x | x | x | x | x |
| Diary | x | x | x | x | x |
| Clinical examinations | | | | | |
| EKG | x | x | x | x | x |
| Echocardiography | x |  |  |  |  |
| Ergometry with lactate measurement | x |  | x |  | x |
| Blood pressure | x | x | x | x | x |
| BMI | x | x | x | x | x |
| Body fat analysis (caliper) | x | x | x | x | x |
| Weight | x | x | x | x | x |
| Laboratory examinations | | | | | |
| (see table 2S) | x | x | x | x | x |

Table 1S: Measurements assessed

| *Assessed laboratory parameters (if available)* | |
| --- | --- |
| **Initial laboratory tests** | **Laboratory tests during intervention period** |
| - Complete differential blood count - FACS: lymphocytes, T-cell subsets, NKC - Serum electrolytes - Liver function parameters - Renal function parameters - Serum albumin - Total serum protein - Iron status, folic acid, vitamin B12 - Lipid status, FFA (free fatty acids) - Leptin - LH, FSH, E2, Pg, Testosterone, SH-BP - TSH, fT3, fT4 - IGF-1, IGF-1-BP - HbA1c, C-peptide - Fasting glucose and insulin - Homocysteine - TNFalpha - CRP - CEA, CA15-3 - PZ - pBNP, CK, CK-MB, Trop-T - Immunoglobulins (quantitative) | - Complete differential blood count - FACS: lymphocytes, T-cell subsets, NKC - Lipid status, FFA (free fatty acids) - LH, FSH, E2, Pg, Testosterone, SH-BP - IGF-1, IGF-1-BP - HbA1c, C-peptide - Fasting glucose and insulin - Leptin - Homocysteine - TNFalpha - CRP - CEA, CA15-3 - Total serum protein - Immunoglobulins (quantitative)   To be measured at 3, 6, 9 and 12 months |

Table 2S: Flowchart of laboratory examinations

|  | | | **Unsupervised training** | | | | **Supervised training** | | | |
| --- | --- | --- | --- | --- | --- | --- | --- | --- | --- | --- |
|  | | months | mean | 95%CI Lower bound | 95%CI Upper bound | *p* | mean | 95%CI Lower bound | 95%CI Upper bound | *p* |
| Blood pressure systolic (mmHg) | Baseline | | 137 | 130.67 | 143.73 | 0.619 | 136 | 128.74 | 142.3 | 0.825 |
|  | 3 | | 133 | 125.89 | 140.57 |  | 133 | 125.12 | 141.45 |  |
|  | 6 | | 133 | 124.67 | 141,.7 |  | 131 | 124.54 | 136.60 |  |
|  | 9 | | 131 | 122.38 | 140.1 |  | 135 | 126.62 | 142.85 |  |
|  | 12 | | 131 | 125.54 | 136.12 |  | 133 | 123.62 | 141.79 |  |
| Blood pressure diastolic (mmHg) | Baseline | | 83.52 | 79.81 | 87.23 | 0.438 | 81.08 | 77.28 | 84.88 | 0.969 |
|  | 3 | | 80.09 | 75.20 | 84.98 |  | 80.14 | 74.96 | 85.32 |  |
|  | 6 | | 79.5 | 75.96 | 83.04 |  | 80.25 | 75.38 | 85.12 |  |
|  | 9 | | 79.33 | 75.72 | 82.95 |  | 81.68 | 77.94 | 85.43 |  |
|  | 12 | | 79.78 | 76.68 | 82.88 |  | 82.12 | 76.55 | 87.69 |  |
| Cholesterin (mg/dl) | Baseline | | 231.75 | 216.42 | 247.08 | 0.620 | 228.12 | 207.61 | 248.63 | 0.941 |
|  | 3 | | 231.77 | 213.15 | 250.4 |  | 237.95 | 214.3 | 261.6 |  |
|  | 6 | | 220.82 | 200.92 | 240.72 |  | 226.05 | 204.7 | 247.41 |  |
|  | 9 | | 226.19 | 206.35 | 246.03 |  | 226.89 | 204.33 | 249.45 |  |
|  | 12 | | 216.2 | 198.57 | 233.84 |  | 227.69 | 198.8 | 256.58 |  |
| HDL (mg/dl) | Baseline | | 71.28 | 61.07 | 81.49 | 0.926 | 72.05 | 62.42 | 81.67 | 0.939 |
|  | 3 | | 72.68 | 62.99 | 82.30 |  | 70.42 | 62.77 | 78.07 |  |
|  | 6 | | 67.79 | 59.78 | 75.8 |  | 70.72 | 61.58 | 79.87 |  |
|  | 9 | | 72.06 | 62.58 | 81.53 |  | 67.35 | 59.61 | 75.1 |  |
|  | 12 | | 70.69 | 58.96 | 82.42 |  | 69.07 | 59.13 | 79.01 |  |
| SHBG (nmol/l) | Baseline | | 60.68 | 48.46 | 72.91 | 0.90 | 63.08 | 49.07 | 77.1 | 0.947 |
|  | 3 | | 55.09 | 45.68 | 64.51 |  | 69.30 | 52.86 | 85.74 |  |
|  | 6 | | 54.56 | 43.04 | 66.09 |  | 68.94 | 51 | 86.87 |  |
|  | 9 | | 52.04 | 37.44 | 66.63 |  | 70.54 | 50.98 | 90.1 |  |
|  | 12 | | 55.64 | 41.72 | 69.56 |  | 71.68 | 44.52 | 98.83 |  |

**Table 3S**: Development of blood pressure and selected laboratory parameters as well as Quality of life over time

|  | | **Unsupervised training** | | | | **Supervised training** | | | |
| --- | --- | --- | --- | --- | --- | --- | --- | --- | --- |
|  | months | mean | 95%CI Lower bound | 95%CI Upper bound | *p* | mean | 95%CI Lower bound | 95%CI Upper bound | *p* |
| Insulin (mclU/ml | Baseline | 10.75 | 8.24 | 13.27 | 0.474 | 16.10 | 4.65 | 27.55 | 0.855 |
|  | 3 | 13.15 | 8.65 | 17.65 |  | 11.50 | 6.69 | 16.31 |  |
|  | 6 | 9.84 | 7.96 | 11.73 |  | 11.50 | 8.17 | 14.84 |  |
|  | 9 | 13.39 | 8.52 | 18.26 |  | 11.15 | 7.89 | 14.41 |  |
|  | 12 | 11.23 | 6.34 | 16.11 |  | 9.90 | 5.86 | 13.94 |  |
| Progesterone (ng/ml) | Baseline | 0.22 | 0.15 | 0.29 | 0.273 | 0.16 | 0.11 | 0.21 | 0.694 |
|  | 3 | 0.31 | 0.19 | 0.42 |  | 0.18 | 0.11 | 0.25 |  |
|  | 6 | 0.28 | 0.18 | 0.38 |  | 0.19 | 0.11 | 0.26 |  |
|  | 9 | 0.35 | 0.17 | 0.53 |  | 0.2 | 0.11 | 0.28 |  |
|  | 12 | 0.36 | 0.22 | 0.51 |  | 0.22 | 0.15 | 0.29 |  |
| IGF1 (ng/ml) | Baseline | 139.31 | 114.56 | 164.07 | 0.440 | 159.78 | 124.15 | 195.41 | 0.167 |
|  | 3 | 148.6 | 104.41 | 192.79 |  | 163.87 | 121.84 | 205.9 |  |
|  | 6 | 164.08 | 133.87 | 194.29 |  | 166.25 | 113.86 | 218.63 |  |
|  | 9 | 151.61 | 124.89 | 178.34 |  | 157.23 | 111.22 | 203.24 |  |
|  | 12 | 134.34 | 113.17 | 155.5 |  | 118.51 | 89.4 | 147.62 |  |
| Body fat | Baseline | 36.40 | 33.22 | 39.58 | 0.978 | 38.68 | 36.34 | 41.03 | 0.989 |
|  | 3 | 36.39 | 32.86 | 39.93 |  | 38.88 | 36.57 | 41.20 |  |
|  | 6 | 37.30 | 34.59 | 40.02 |  | 38.39 | 35.8 | 40.98 |  |
|  | 9 | 36.22 | 32.44 | 39.99 |  | 38.67 | 36.08 | 41.26 |  |
|  | 12 | 37.15 | 34.33 | 39.97 |  | 38.06 | 35.62 | 40.50 |  |
| Physical function | Baseline | 86.36 | 80.08 | 92.65 | 0.288 | 88.26 | 82.10 | 94.43 | 0.939 |
|  | 3 | 89.7 | 85.07 | 94.32 |  | 87.3 | 82.05 | 92.55 |  |
|  | 6 | 89.86 | 85.43 | 94.28 |  | 88 | 80.66 | 95.34 |  |
|  | 9 | 93.94 | 89.88 | 98 |  | 85.61 | 78.1 | 93.13 |  |
|  | 12 | 90.3 | 84.55 | 96.05 |  | 89.58 | 83.1 | 96.07 |  |
| Role function | Baseline | 72.73 | 61.66 | 83.79 | 0.470 | 81.94 | 70.59 | 93.3 | 0.976 |
|  | 3 | 81.06 | 75.34 | 86.78 |  | 82.54 | 71.95 | 93.13 |  |
|  | 6 | 79.71 | 69.76 | 89.66 |  | 83.33 | 71.46 | 95.20 |  |
|  | 9 | 85.71 | 74.65 | 96.78 |  | 78.07 | 64.4 | 91.74 |  |
|  | 12 | 84.09 | 73.28 | 94.9 |  | 80.21 | 65.99 | 94.43 |  |

**Table 4S**: Development of blood pressure and selected laboratory parameters as well as Quality of life over time
